# The diagnostic accuracy of superb microvascular imaging in distinguishing thyroid nodules: A protocol for systematic review and meta analysis

**DOI:** 10.1097/MD.0000000000022350

**Published:** 2020-10-02

**Authors:** Congliang Tian, Zinan Wang, Xiukun Hou, Cong Wang

**Affiliations:** a Pediatrics Department of the First Affiliated Hospital of Dalian Medical University; b Nutrition Department of the First Affiliated Hospital of Dalian Medical University; c Ultrasound Department of the First Affiliated Hospital of Dalian Medical University, Dalian City, Liaoning Province, China.

**Keywords:** meta-analysis, superb microvascular imaging, thyroid nodule

## Abstract

**Background:**

Ultrasonography is the first choice for clinical diagnosis and differentiation of thyroid cancer Currently. However, due to the complexity and overlapping nature of the thyroid nodule sonograms, it remains difficult to accurately identify nodules with atypical ultrasound characteristics. Previous studies showed that superb microvascular imaging (SMI) can detect tumor neovascularization to differentiate benign from malignant thyroid nodules. However, the results of these studies have been contradictory with low sample sizes. This meta-analysis tested the hypothesis that SMI is accurate in distinguishing benign and malignant thyroid nodules.

**Methods:**

We will search PubMed, Web of Science, Cochrane Library, and Chinese biomedical databases from their inceptions to the August 20, 2020, without language restrictions. Two authors will independently carry out searching literature records, scanning titles and abstracts, full texts, collecting data, and assessing risk of bias. Review Manager 5.2 and Stata14.0 software ((Stata Corp, College Station, TX) will be used for data analysis.

**Results:**

This systematic review will determine the accuracy of SMI in distinguishing thyroid nodules.

**Conclusion:**

Its findings will provide helpful evidence for the accuracy of SMI in in distinguishing thyroid nodules.

Systematic review registration: INPLASY202080084

## 1 Introduction

Thyroid cancer is a common malignant disease that accounts for about 1% of all cancer patients.^[1]^ Solid thyroid nodules are a risk factor for thyroid cancer, and it is critical to accurately differentiate thyroid nodules.^[2]^ Currently, ultrasonography is the first choice for clinical diagnosis and differentiation of thyroid cancer.^[3]^ However, due to the complexity and overlapping nature of the thyroid nodule sonograms, it remains difficult to accurately identify nodules with atypical ultrasound characteristics.^[4]^

Benign and malignant thyroid nodules display variation in blood flow patterns and vascular morphology that are useful in separating one from the other.^[5]^ Color Doppler flow imaging can show blood flow inside a tumor, but is not effective at imaging some low-velocity microvessels.^[6]^ Superb microvascular imaging (SMI) is a novel ultrasonic technique that can quickly, simply, and noninvasively monitor the microvascular distribution in a tumor and evaluate microvascular perfusion.^[7]^ SMI uses a multidimensional filter to eliminate extraneous signal while preserving low-velocity flow signals. In contrast, conventional Doppler systems use a single-dimension filter and are inadequate in distinguishing low-velocity flow signals that overlap from background.^[8]^ Previous studies showed that SMI can detect tumor neovascularization to differentiate benign from malignant thyroid nodules.^[9]^ However, the results of these studies have been contradictory with low sample sizes. Therefore, this meta-analysis aimed to determine the accuracy of SMI for the differential diagnosis of benign and malignant thyroid nodules.

## 2 Materials and methods

This study was conducted in accordance with the PRISMA (Preferred Reporting Items for Systematic Reviews and Meta Analyses) guidelines and the protocol was registered in the INPLASY (INPLASY202080084).

### 2.1 Eligibility criteria

#### 2.1.1 Type of study

This study will only include high quality clinical cohort or case control studies.

#### 2.1.2 Type of patients

The patients should be those who had undergone thyroid nodule.

#### 2.1.3 Intervention and comparison

This study will compare SMI with pathology for diagnosing thyroid nodules.

#### 2.1.4 Type of outcomes

The primary outcomes include sensitivity, specificity, positive, and negative likelihood ratio, diagnostic odds ratio, and the area under the curve of the summary receiver operating characteristic.

### 2.2 Search methods

PubMed, Web of Science, Cochrane Library, and Chinese biomedical databases will be searched from their inceptions to the August 20, 2020, without language restrictions. The search strategy for PubMed is shown in Table 1. Other online databases will be used in the same strategy.

**Table 1:**
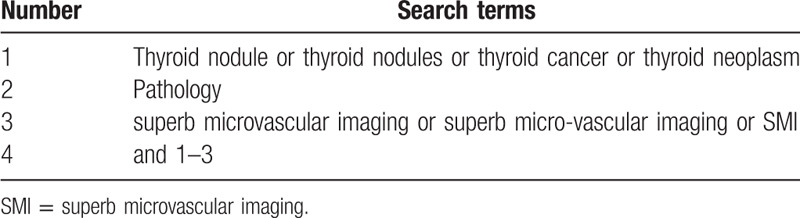
Search strategy sample of PubMed.

### 2.3 Data extraction and quality assessment

Two authors will independently select the trials according to the inclusion criteria, and import into Endnote X9 (Thomson Corporation, Stanford, USA). Then remove duplicated or ineligible studies. Screen the titles, abstracts, and full texts of all literature to identify eligible studies. All essential data will be extracted using previously created data collection sheet by 2 independent authors. Discrepancies in data collection between 2 authors will be settled down through discussion with the help of another author. The following data will be extracted from each included research: the first author's surname, publication year, language of publication, study design, sample size, number of lesions, source of the subjects, instrument, “gold standard,” and diagnostic accuracy. The true positives, true negatives, false positives, and false negatives in the fourfold (2 x 2) tables were also collected. Methodological quality was independently assessed by 2 researchers based on the quality assessment of studies of diagnostic accuracy studies (QUADAS) tool. The QUADAS criteria included 14 assessment items. Each of these items was scored as “yes” (2), “no” (0), or “unclear”(1). The QUADAS score ranged from 0 to 28, and a score ≥22 indicated good quality. Any disagreements between 2 investigators will be solved through discussion or consultation by a 3rd investigator.

### 2.4 Statistical analysis

The STATA version 14.0 (Stata Corp, College Station, TX) and Meta-Disc version 1.4 (Universidad Complutense, Madrid, Spain) softwares were used for meta-analysis. We calculated the pooled summary statistics for sensitivity, specificity, positive and negative likelihood ratio, and diagnostic odds ratio with their 95% confidence intervals. The summary receiver operating characteristic curve and corresponding area under the curve were obtained. The threshold effect was assessed using Spearman correlation coefficients. The Cochran's Q-statistic and I test were used to evaluate potential heterogeneity between studies. If significant heterogeneity was detected (Q test *P* < .05 or I test>50%), a random effects model or fixed effects model was used. We also performed sub group and meta-regression analyses to investigate potential sources of heterogeneity. To evaluate the influence of single studies on the overall estimate, a sensitivity analysis was performed. We conducted Begg funnel plots and Egger linear regression tests to investigate publication bias.

### 2.5 Ethics and dissemination

We will not obtain ethic documents because this study will be conducted based on the data of published literature. We expect to publish this study on a peer-reviewed journal.

## 3 Discussion

Thyroid nodules are a common finding, and their accurate differentiation is important for clinical decision-making. High-resolution ultrasound plays an important role in the differential diagnosis of thyroid nodules.^[10]^ The ultrasound features of thyroid malignant nodules include a low echo, unclear margin, microcalcification, and aspect ratio > 1. These findings increase the likelihood that a nodule is malignant. However, no single ultrasound feature can independently diagnose malignant nodules.^[11]^ The blood flow distribution patterns of benign and malignant thyroid nodules are different.^[12]^ Blood vessels and flow characteristics in thyroid nodules have been employed in the differential diagnosis of benign and malignant thyroid nodules[^13^,^14^]; however, the value of color Doppler flow patterns in the diagnosis of benign and malignant thyroid nodules remains controversial. SMI uses high-resolution Doppler technology (Aplio diagnostic equipment) to build a high-density beamformer. Traditional Doppler ultrasound uses filtering to eliminate noise and motion artifacts resulting in a loss of low-speed blood flow information. SMI technology can identify the noise generated by blood flow and tissue movement and uses adaptive calculation methods to display the real blood flow data.^[8]^ This work will systematically evaluate the technical performance and accuracy of SMI for differential diagnosis of benign and malignant thyroid nodules.

## Author contributions

**Conceptualization:** Cong Wang, Xiukun Hou.

**Data curation:** Congliang Tian, Zinan Wang, Cong Wang.

**Methodology:** Congliang Tian, Zinan Wang.

**Writing – original draft:** Xiukun Hou.

**Writing – review & editing:** Xiukun Hou.
